# Excitation and Tuning of Fano-like Resonances in Whispering Gallery Microcavity and Microfiber Modal Interferometer Coupled System

**DOI:** 10.3390/s26123644

**Published:** 2026-06-07

**Authors:** Qihao Yang, Hongrong Zheng, Weihan Zhang, Xiaoming Zhang, Zhenyu Wang, Yifei Han, Biqiang Jiang

**Affiliations:** Key Laboratory of Light Field Manipulation and Information Acquisition, Ministry of Industry and Information Technology, Northwestern Polytechnical University, Xi’an 710129, China; yamg@mail.nwpu.edu.cn (Q.Y.); hrzheng@mail.nwpu.edu.cn (H.Z.); zhangweihan@mail.nwpu.edu.cn (W.Z.); xmzhang@mail.nwpu.edu.cn (X.Z.); 2023265156@mail.nwpu.edu.cn (Z.W.); hyff@mail.nwpu.edu.cn (Y.H.)

**Keywords:** Fano-like resonance, whispering gallery mode (WGM) microcavity, microfiber modal interferometer (MMI), Fano-like lineshape tuning

## Abstract

We propose a method for the excitation and controllable tuning of Fano-like resonance based on whispering gallery mode (WGM) microcavities and microfiber modal interferometers (MMIs). By the interaction of the discrete comb-like resonant modes excited by the WGM microcavity and the continuous interference spectrum generated by the MMI, the excitation of Lorentzian, Fano-like resonance, and electromagnetically induced transparency (EIT) lineshapes is achieved. In this system, the resonant modes of thin-walled WGM can interact with the liquid inside the cavity; thus, the Fano-like lineshape can be tuned via intracavity refractive index modulation. By adjusting the diameter and transition region length of the MMI, the Fano-like lineshape generated by the WGM-MMI coupled structure can be tuned. More importantly, as the refractive index of the liquid inside the cavity increases from 1.33 to 1.351, the Fano-like resonance lineshape evolves and the corresponding Fano parameter *q* shifts from 0.19 to 1.24. The proposed system enables stable excitation and controllable tuning of Fano-like resonances, demonstrating potential for applications in microfluidic sensing and optical switching.

## 1. Introduction

Fano resonance arises from the quantum interference between a discrete excited state of an atom and a continuum with the same energy level [1,2], where the absorption at the discrete resonance results in its asymmetric lineshape. Analogous phenomena can also be observed in optics. The phase difference between the discrete and continuous modes shifts accordingly, generating various types of Fano-like resonance lineshapes. Generally, the Fano parameter *q* is utilized to describe various Fano-like profiles. It determines the asymmetric slope of the lineshape, indicating the degree of drastic changes in optical transmittance over an ultra-narrow wavelength range. This distinctive characteristic enables the application of Fano-like resonances in a variety of photonic devices, such as optical sensors [3,4,5], optical switches [6,7,8], and lasers [9,10,11,12].

Currently, Fano-like resonance has been observed in various photonic structures, including photonic crystals [13,14,15], surface plasmon polariton (SPP) structures [16,17,18] all-fiber structures [19,20,21,22] and microresonators. Due to fixed structural parameters, photonic crystals face challenges in achieving tunability of Fano-like lineshapes. And the large resonance bandwidth commonly observed in SPP-based and many other all-fiber structures constrains the limit of detection (LOD) of Fano-like resonances in high-sensitivity sensing applications. The Fano-like resonance excitation method based on microresonators typically involves coupling a single resonator or multiple resonators with microfiber [23,24,25]. Light is coupled into the microresonator via the evanescent field of a microfiber. This method offers the advantages of high coupling efficiency, narrow bandwidth, and flexible tunability.

Among existing optical resonators, whispering gallery mode (WGM) microcavities confine light through continuous total internal reflection, featuring high *Q* factors and small mode volumes [26,27,28,29]. WGM microcavities provide ideal platforms for generating discrete resonant modes and have been widely utilized in the excitation of Fano-like resonances. By incorporating a WGM resonator into one arm of a Mach–Zehnder interferometer (MZI) [30], Fano-like resonances can be excited and tuned. However, conventional MZI is highly susceptible to environmental perturbations, resulting in unstable interference spectra.

To solve this issue, we propose a coupled structure comprising a thin-walled WGM microcapillary and a microfiber modal interferometer (MMI) to excite Fano-like resonances. In this configuration, the thin-walled WGM microcapillary provides the discrete resonant modes, while the interference spectrum generated by the MMI serves as the continuous mode. The thin-walled WGM microcapillary is fabricated from a fused silica tube using a rheological forming method [31]. And the MMI is fabricated via a two-step tapering method [32], featuring a large taper angle that induces interference between the fundamental and higher-order modes, thereby generating a highly stable interference spectrum. Based on this coupled structure, multiple Fano-like resonances can be obtained. Finally, by injecting glucose solutions of varying concentrations into the microcavity, these Fano-like resonances can be tuned.

## 2. Theory and Model

**Figure 1:**
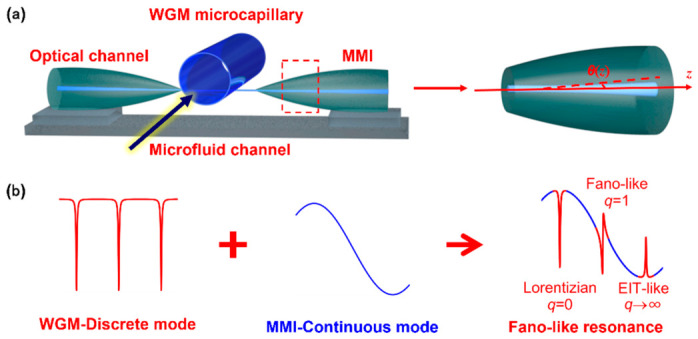
(**a**) Schematic diagram of the WGM-MMI coupled structure; (**b**) schematic illustration of the excitation principle for Fano-like resonances.

Figure 1a illustrates the structure and the operating principle of the WGM-MMI coupled system for the excitation and tuning of Fano-like resonances. A thin-walled WGM microcapillary, featuring a discrete comb-like spectrum, is coupled with a MMI that provides a continuous interference spectrum. By precisely optimizing the coupling position and angle, an ideal Fano-like resonance spectrum can be achieved. Given that the wall of the WGM microcapillary is sufficiently thin, the WGM resonant modes can effectively interact with the liquid confined within the cavity. Consequently, by filling the microcavity with liquids of varying refractive indices, the phase difference between the discrete and continuum modes can be systematically modulated, thereby realizing the dynamic tuning of the Fano-like resonances.

The dense comb-like spectrum generated by the WGM microcapillary provides multiple discrete resonances which enable the simultaneous generation of Lorentzian, Fano-like, and electromagnetically induced transparency (EIT) lineshapes. Compared with the symmetric Lorentzian and EIT lineshapes, the Fano-like lineshape exhibits pronounced asymmetry. To precisely characterize the Fano-like resonance profiles, the Fano parameter *q* is introduced. The absolute value of *q* denotes the intensity ratio of the discrete mode to the continuum mode. Consequently, distinct Fano-like resonance lineshapes correspond to different *q* values, which can be calculated using the following equation:

(1) $$I(\lambda )=D^{2}\frac{(q+\Omega {)}^{2}}{1+\Omega^{2}}$$

where *I* denotes the optical intensity, *q* = cot *δ* is Fano parameter, and *δ* represents the phase difference between the continuum and discrete modes. The other variables are defined as *D*^2^ = 4 sin^2^ *δ* and *Ω* = 2(*λ* − *λ*_c_)/Δ*λ*, where *λ*_c_ and Δ*λ* correspond to the central wavelength and the linewidth of the microcavity resonance peak, respectively. As depicted in the schematic in Figure 1b, the Fano parameter *q* of typical Lorentzian, Fano-like and EIT-like lineshapes correspond to 0, ±1 and ∞.

## 3. Methodology

Due to its intrinsic microfluidic channel, the WGM microcapillary can be filled with liquids of different refractive indices, thereby enabling the modulation of the Fano-like lineshape without altering the coupled structure. However, in a microcapillary with a wall thickness of several tens of micrometers, the electromagnetic field is tightly confined within the wall itself, making it difficult to interact with the liquid inside the microcapillary and limiting spectral modulation capability. Reducing the wall thickness can manipulate the WGM field profile [33], shifting its peak intensity from the cavity wall toward the inner surface. This enhanced field interacting with the intracavity fluid significantly improves spectral modulation capability.

**Figure 2:**
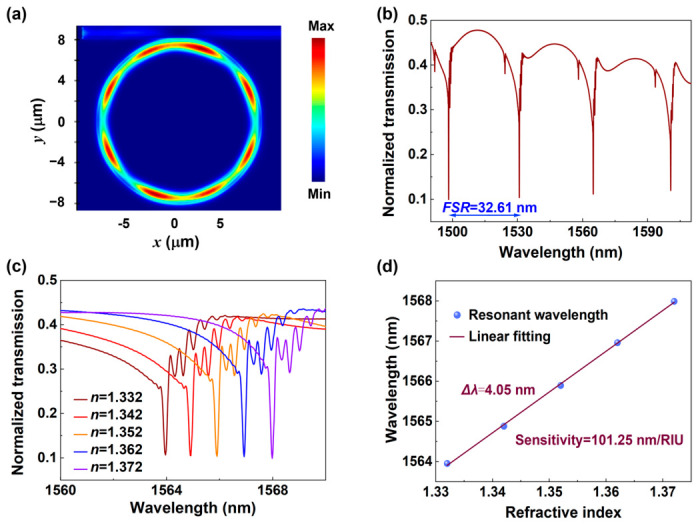
Numerical simulations of a WGM microcapillary with a wall thickness of 1 μm: (**a**) spatial distribution of the optical field at the resonant wavelength; (**b**) transmission spectrum; (**c**) variations in transmission spectra under different refractive index environments within the microcavity; (**d**) refractive index sensitivity.

We first employed the finite-difference time-domain (FDTD) method to establish a coupling model including a WGM microcapillary with 1-μm-thick wall and a microfiber with a 1 μm diameter. To reduce computational memory consumption, a scaled-down WGM microcapillary model with a diameter of 16 μm and appropriate mesh resolution are employed in the simulation. It should be noted that the purpose of this simulation is not to quantitatively reproduce the experimental spectra, but rather to qualitatively reveal the physical mechanism of WGM and the effect of the thin-walled microcapillary on the intracavity refractive index. Therefore, compared with the experimental results, the simulated results show discrepancies in parameters such as the *FSR*, extinction ratio, and *Q* factor. The simulated optical field distribution is illustrated in Figure 2a. At the resonant wavelength of 1594.2 nm, the pump light is coupled into the microcavity, forming a WGM resonance; thus, the majority of the optical field is tightly confined within the microcavity. The normalized output spectrum is presented in Figure 2b, where four distinct resonant peaks appear within the 1500–1600 nm range, with an *FSR* of approximately 32.5 nm. It can be observed that, in addition to the resonant peaks with high extinction ratios, the transmission spectrum also contains accompanying side lobes. These side lobes represent higher-order modes excited by the coupling between the microfiber and the microcavity.

To simulate the refractive index response of the proposed device, the refractive index of the environment inside the microcapillary was incrementally increased from 1.332 to 1.372 in steps of 0.01. The resulting spectral response is shown in Figure 2c, demonstrating a total redshift of 4.05 nm for the resonant peaks. As derived and depicted in Figure 2d, the corresponding refractive index sensitivity of this microcavity reaches 101.25 nm/RIU.

In the experiment, a rheological drawing technique was employed to fabricate the thin-walled WGM microcapillary. A fused silica capillary TSP250350 (Polymicro Technologies, Lisle, IL, USA) with outer diameter of 322 μm and inner diameter of 250 μm was selected. Compared to other fused silica tubes, TSP250350 features a larger inner-to-outer diameter ratio, thereby facilitating the fabrication of thin-walled WGM microcapillary.

During the fabrication process, one end of the fused silica tube was first sealed. Nitrogen gas was then injected into the other end to increase the internal pressure. Once the pressure reached an optimal level, the gas valve was closed to seal the internal cavity. Subsequently, a hydrogen–oxygen flame was applied to heat the tube, causing the heated region to rapidly soften into a molten state. During this process, the sealed gas underwent thermal expansion, while the tube was simultaneously drawn in opposite directions by two motorized translation stages. Driven by the common effect of the internal pressure and the drawing force, the molten silica tube exhibited controllable radial and axial deformations, ultimately generating a uniform WGM microcapillary with a large diameter and a thin wall. Microcapillaries fabricated using this method possess a very smooth outer surface and demonstrate a quality *Q* factor exceeding 10^3^.

**Figure 3:**
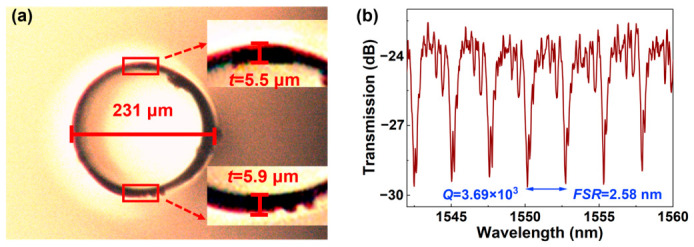
(**a**) Optical microscopy images of the end face of thin-walled WGM microcapillary; (**b**) transmission spectrum of thin-walled WGM microcapillary.

The cross-sectional optical micrograph of the fabricated microcapillary is presented in Figure 3a, revealing an outer diameter of 231 μm. The upper and lower wall thicknesses are 5.5 μm and 5.9 μm respectively, which are significantly reduced compared to the initial fused silica tube. The slight difference between the upper and lower wall thicknesses is attributed to the gravity acting on the microcapillary while in the molten state.

Furthermore, we characterized the transmission spectrum of this microcapillary, as depicted in Figure 3b. The measured *Q* factor is 3.69 × 10^3^, with an *FSR* of 2.58 nm. Compared with some microspheres with ultrahigh *Q* factor, the thin-walled microcapillary fabricated in our work exhibits a relatively lower *Q* factor, which is mainly related to its geometric structure and fabrication process. First, a thinner wall significantly weakens the optical confinement capability of the microcapillary, allowing a large portion of the optical field to leak out of the resonator and thereby reduce the *Q* factor. Second, a microsphere can provide strong three-dimensional optical confinement, whereas a microcapillary can only confine light effectively in two dimensions; the optical confinement along the *z*-axis is much weaker, and therefore its *Q* factor is generally lower than that of a microsphere. In addition, surface roughness, geometric nonuniformity, and extra scattering losses introduced during fabrication can also lead to a reduction in the *Q* factor. Compared with other reported work on thin-walled microcapillary, the *Q* factor of our fabricated microcapillary is comparable to theirs (approximately 6 × 10^3^) [31].

Compared to the unprocessed WGM microcapillary, its spectral modulation capability is substantially enhanced. Due to dust attaching to the inner wall of the microcapillary, several higher-order modes with low extinction ratios appear in the WGM transmission spectrum. By further optimizing fabrication parameters such as the internal pressure, drawing velocity, and elongation distance, the wall thickness of the fabricated microcapillary can be reduced down to 3 μm.

To generate interference spectra within the microfiber, coupling between the propagating fundamental mode and higher-order modes must be generated. In the microfiber, the taper angle of the transition region is defined as *θ*(*z*) = tan^−1^(d*ρ*/d*z*), where *ρ* represents the core radius within the transition region and *z* denotes the axial length of the fiber. When the taper angle in the transition region is sufficiently large, coupling occurs between the distinct modes propagating within the fiber, leading to inter-modal interference.

**Figure 4:**
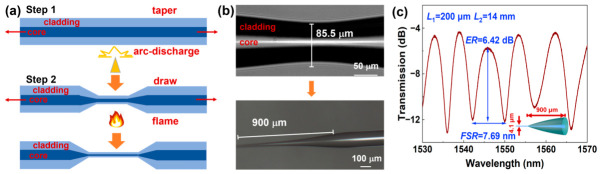
(**a**) Schematic diagram of the process for fabricating MMI by the two-step tapering method; (**b**) optical microscopy images of the fabricated MMI; (**c**) interference spectrum of MMI.

We employed a two-step drawing technique to fabricate the MMI. As illustrated in Figure 4a, a single mode fiber was first pre-treated utilizing the taper splicing function of a fusion splicer. During taper splicing, there is simultaneous movement of the two internal motors in opposite directions during the electrode arc discharge, thereby inducing a biconical transition structure at the splicing point. In our experiment, the drawing length for this taper splicing stage was configured to 200 μm (*L*_1_). As shown in Figure 4b, an initial taper angle was formed at this junction, with the diameter of the tapered region reducing to 85.5 μm. Subsequently, the hydrogen–oxygen flame-brushing technique was applied for further tapering. Typically, to enhance the coupling efficiency between the MMI and the WGM microcavity, the waist diameter of the MMI is kept below 5 μm. Therefore, the elongation length was set to 14 mm (*L*_2_). As illustrated in Figure 4b,c, the prepared MMI has a transition region length of only 900 μm and a diameter of 4.1 μm. The interference spectrum of the MMI exhibits an extinction ratio of 6.42 dB and a *FSR* of 7.69 nm.

## 4. Results and Discussions

In the experiment, the fabricated MMI was placed on a low-refractive-index Teflon substrate. This substrate not only preserves the optical waveguide properties and inter-modal interference effects of the MMI but also enhances the overall structural stability of the device. Meanwhile, the thin-walled WGM microcapillary was fixed into a three-dimensional translation stage. The employed three-dimensional translation stage facilitated precise manipulation of the coupling position and angle between the microcapillary and the MMI. As the microcapillary is translated along the axial direction of the microfiber, the local diameter of the microfiber varies depending on the specific coupling position. Consequently, this variation alters the phase difference between the fundamental mode and the higher-order modes, leading to corresponding changes in the extinction ratio and lineshape of the excited Fano-like resonances. In our experimental configuration, the microcapillary was brought into perpendicular contact with the interferometer.

Utilizing the aforementioned two-step tapering method, the taper splicing length was fixed at 200 μm. By setting different hydrogen–oxygen flame tapering length, the fabricated MMIs achieved diameters of 3.9, 2.6, and 2.4 μm, respectively. Since MMI with distinct diameters support different higher-order modes, the interference spectra vary accordingly. Consequently, the coupling strength and phase difference between the WGM microcavity and the MMI also vary, enabling the observation of Fano-like resonance spectra characterized by diverse Fano parameters *q*.

To generate multiple Fano-like resonances within a single interference cycle, an index-matching liquid with a refractive index of 1.34 (Cargille, Cedar Grove, NJ, USA) was employed to fully immerse the coupling region. The changed local refractive index environment leads to increases in both the extinction ratio and the *FSR* of the interference spectrum, thereby allowing simultaneous excitation of multiple Fano-like resonances.

**Figure 5:**
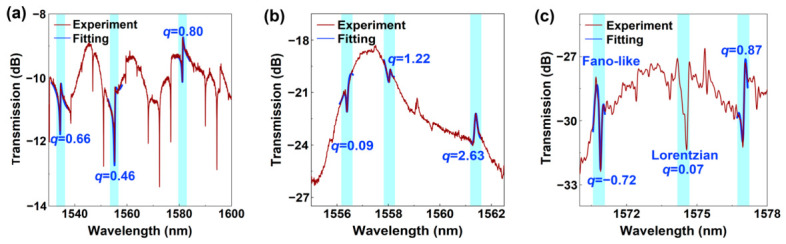
Lorentzian, Fano-like and EIT lineshapes for MMIs with different diameters *d*: (**a**) *d* = 3.9 μm; (**b**) *d* = 2.6 μm; (**c**) *d* = 2.4 μm. The blue region in the inset represents the fitting region.

As illustrated in Figure 5a–c, the three fabricated MMIs were individually coupled with a WGM microcapillary. Figure 5a shows the Fano-like resonance spectra generated by coupling an MMI with a diameter of 3.9 μm with a WGM microcapillary. Multiple Fano-like lineshapes were observed over a broad wavelength range of 1530–1600 nm. However, both the interference spectrum and the Fano-like resonances exhibited relatively low extinction ratios. As the MMI diameter was reduced to 2.6 μm and 2.4 μm, respectively, as shown in Figure 5b,c, coupling between the fundamental mode and higher-order modes became more efficient, resulting in an increased extinction ratio of the interference spectrum. In addition, as the intensities of the continuous and discrete modes became more comparable, the extinction ratio of the Fano-like resonance was further enhanced.

We further analyzed the variation in the Fano parameter *q*. According to the Fano formula, we fitted the different Fano-like lineshapes to obtain the corresponding Fano parameter *q*. As shown in Figure 5b, the coupled lineshape reaches a maximum Fano parameter of *q* = 2.63, corresponding to a symmetric EIT profile. As shown in Figure 5c, when *q* = 0.87, which is close to 1, the coupled lineshape exhibits a typical asymmetric Fano-like profile. When *q* = 0.07, which is close to 0, the coupled lineshape exhibits a symmetric Lorentzian profile. In addition, the spectrum exhibits a maximum extinction ratio of 4.33 dB and a maximum slope of 36.63 dB/nm. This slope is higher than those reported in some early microcavity-based Fano-like resonance systems, although it is still lower than that achieved in some recently reported on-chip microresonator platforms. The slope of the Fano-like lineshape is mainly limited by the *Q* factor of the microcapillary. If the *Q* factor can be further improved, the Fano-like lineshape is expected to exhibit a higher slope, a higher extinction ratio, and a narrower full width at half maximum.

The extracted Fano parameter *q* of the excited Fano-like resonances varies from −0.72 to 2.63, spanning Lorentzian, Fano-like, and EIT lineshapes. Compared with related reported works, this tuning range of *q* is relatively broad. We attribute this to the large tunable range of the phase difference Δ*φ*, which can be achieved by adjusting the diameter of the MMI.

To further realize refractive-index tuning of the Fano-like resonance within a single device, the fabricated WGM microresonator was coupled to an MMI and filled with liquids of varying refractive indices, thereby introducing an additional phase difference Δ*φ*, between the discrete and continuous modes. For the experiment, distilled water and three distinct glucose solutions were prepared. The corresponding refractive indices were 1.330, 1.337, 1.344, and 1.351, respectively. During the measurements, one end of the fabricated thin-walled WGM microcapillary was immersed in the liquid sample, while the opposite end was connected to a micro-syringe.

**Figure 6:**
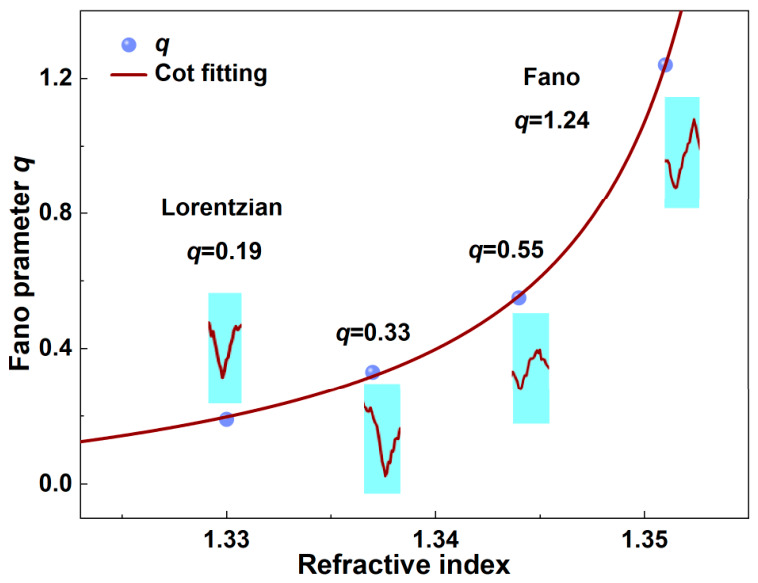
Evolution of the Fano-like lineshape at 1584.5 nm and the corresponding cotangent fit of Fano parameter *q*. The inset shows the blue region used for fitting and the Fano-like lineshape.

During liquid exchange, the microcapillary was secured with a UV adhesive to maintain a stable WGM-MMI coupling position, thereby preventing structural modification from altering the overall Fano-like resonance profile. As shown in Figure 6, at the resonance wavelength of 1584.5 nm, the initial spectrum exhibits a symmetric Lorentzian lineshape, with a corresponding Fano parameter *q* of 0.19, which is close to 0. As the refractive index of the intracavity liquid increases, the phase difference between the WGM resonance and the interference mode changes. This phase difference determines the evolution of the lineshape into an asymmetric Fano-like profile, with the corresponding Fano parameter *q* increasing to 1.24, which is close to unity. Furthermore, the evolution of the Fano parameter *q* follows a cotangent-function dependence.

These results demonstrate that refractive index modulation of the intracavity liquid can effectively tune the phase difference between the discrete WGM resonance and the MMI mode, without adjusting the coupling position, thereby realizing an evolution from a Lorentzian lineshape to a Fano-like lineshape within a single device.

Compared with other tuning methods for Fano-like lineshapes, the intracavity refractive index tuning method used in this work offers several practical advantages. Unlike some conventional schemes that tune the refractive index of the surrounding the coupling region, our structure prevents direct contact between the microfiber and the index-matching liquid, while the two ends of the microcapillary are fixed. This helps maintain a stable coupling position during tuning. Compared with thermal tuning, the proposed method does not require additional heating components. More importantly, thermal instability makes it challenging to achieve continuous and controllable tuning of the Fano-like lineshape. Compared with strain tuning, our structure does not rely on mechanical deformation and therefore reduces the potential risk of mechanical damage to the thin-walled microcapillary. Compared with coupling adjustment, our method does not require repeated adjustment using a high-precision translation stage, which simplifies the operation and improves repeatability.

Overall, the proposed WGM-MMI structure enables stable and controllable tuning of the Fano-like lineshape without altering the coupling condition, and without the additional heating or stress-applying components. Moreover, this approach is compatible with microfluidic platforms, which makes it promising for tunable photonic devices and optofluidic sensing applications.

## 5. Conclusions

In this study, we propose and experimentally demonstrate a novel method for the excitation and dynamic tuning of Fano-like resonances based on the coupling between a MMI and a thin-walled WGM microcapillary. By comprehensively optimizing the taper waist diameter of the MMI, the lineshape of the Fano-like resonances can be effectively tuned, thereby spanning Lorentzian, standard Fano-like, and EIT lineshapes, with a maximum slope of 36.63 dB/nm. More importantly, through benefiting from the ultra-thin wall of the microcapillary, as the refractive index of the internal liquid varies from 1.330 to 1.351, continuous and controllable tuning of the Fano-like resonance lineshapes can be achieved without altering the coupled structure. The lineshape smoothly evolves from a Lorentzian profile into a standard Fano-like resonance, with the Fano parameter *q* shifting from 0.19 to 1.24. This directly substantiates the considerable application potential of the proposed system in spectral modulation and microfluidic refractive index sensing. Therefore, the proposed WGM-MMI platform provides a compact, flexibly tunable, and highly microfluidic-compatible all-fiber Fano resonance platform, presenting a novel solution for developing integrated optical sensors and optical switches.

## Data Availability

The data presented in this study are available on request from the corresponding author.
